# Assessing mental stress from the photoplethysmogram: a numerical study

**DOI:** 10.1088/1361-6579/aabe6a

**Published:** 2018-05-15

**Authors:** Peter H Charlton, Patrick Celka, Bushra Farukh, Phil Chowienczyk, Jordi Alastruey

**Affiliations:** 1 Department of Biomedical Engineering, School of Biomedical Engineering and Imaging Sciences, King’s College London, King’s Health Partners, St Thomas’ Hospital, London, SE1 7EH, United Kingdom; 2 Polar Electro Oy, Professorintie 5, 90440 Kempele, Finland; 3 Department of Clinical Pharmacology, King’s College London, King’s Health Partners, St Thomas’ Hospital, London, SE1 7EH, United Kingdom; peter.charlton@kcl.ac.uk

**Keywords:** biomedical signal processing, photoplethysmogram, mental stress, 1D modelling, pulse wave propagation

## Abstract

*Objective*: Mental stress is detrimental to cardiovascular health, being a risk factor for coronary heart disease and a trigger for cardiac events. However, it is not currently routinely assessed. The aim of this study was to identify features of the photoplethysmogram (PPG) pulse wave which are indicative of mental stress. *Approach*: A numerical model of pulse wave propagation was used to simulate blood pressure signals, from which simulated PPG pulse waves were estimated using a transfer function. Pulse waves were simulated at six levels of stress by changing the model input parameters both simultaneously and individually, in accordance with haemodynamic changes associated with stress. Thirty-two feature measurements were extracted from pulse waves at three measurement sites: the brachial, radial and temporal arteries. Features which changed significantly with stress were identified using the Mann–Kendall monotonic trend test. *Main results*: Seventeen features exhibited significant trends with stress in measurements from at least one site. Three features showed significant trends at all three sites: the time from pulse onset to peak, the time from the dicrotic notch to pulse end, and the pulse rate. More features showed significant trends at the radial artery (15) than the brachial (8) or temporal (7) arteries. Most features were influenced by multiple input parameters. *Significance*: The features identified in this study could be used to monitor stress in healthcare and consumer devices. Measurements at the radial artery may provide superior performance than the brachial or temporal arteries. *In vivo* studies are required to confirm these observations.

## 1. Introduction

Mental stress is detrimental to cardiovascular health (Brotman *et al* 2007). Acute stressors, such as disasters and family deaths, can trigger cardiac death (Leor *et al* 1996, Kales *et al* 2007, Dimsdale 2008). Chronic stressors, such as job strain, low income level and marital unhappiness, have been found to be a risk factor for coronary heart disease (Rosengren *et al* 1991, Greenwood *et al* 1996, Iso *et al* 2002, Rosengren *et al* 2004, Dimsdale 2008). Both chronic and acute stressors have been associated with elevated blood pressure (Schnall *et al* 1990, Kario *et al* 1997), as well as reduced immune function and susceptibility to respiratory infection (Herbert and Cohen 1993, Pedersen *et al* 2010). The extensive effects of mental stress on cardiovascular health provide great motivation for developing techniques to identify stress, and subsequently treat it (Dimsdale 2008).

A potentially effective and convenient method for assessing mental stress is to extract an index of stress from the photoplethysmogram (PPG) (Abbod *et al* 2011, Singh *et al* 2011, Yoo and Lee 2011, Zheng *et al* 2016, Zangróniz *et al* 2018). The PPG signal is a valuable source of physiological information, since it is influenced by the cardiac, vascular and autonomic nervous systems, which are all affected by stress (Allen 2007). For instance, changes to parameters such as heart rate, blood pressure, and heart rate variability would be expected to influence the PPG signal. In addition, the PPG is easily acquired using pulse oximeters, which are frequently used in healthcare to measure arterial blood oxygen saturation and pulse rate. Furthermore, the PPG can be acquired by a wide range of ubiquitous devices such as smartphones, tablets, and fitness devices (Charlton *et al* 2018). If it was possible to extract a measure of mental stress from the PPG then it may have great utility.

The primary aim of this study was to identify features of the PPG pulse wave which are indicative of mental stress. Secondary aims were to compare different PPG measurement sites for assessing stress, and to analyse the physiological determinants of features which changed with stress. The study was performed using a novel approach to simulate PPG pulse waves numerically at different levels of stress.

## 2. Methods

### 2.1. Simulating PPG pulse waves

Numerical models have been widely used to simulate pulse wave propagation, typically providing simulations of blood flow, blood pressure, and vessel area pulse waves (Shi *et al* 2011). However, these are rarely used to simulate the PPG pulse wave. We now present a novel approach for simulating the PPG using a model of pulse wave propagation.

We used the one-dimensional (1D) formulation of pulse wave propagation to simulate blood pressure signals (Alastruey *et al* 2012). The model consists of a periodic inflow boundary condition modelling flow from the left ventricle into the aortic root; arterial segments modelling the larger arteries as thin, impermeable, deformable cylindrical tubes of constant length and linearly tapered diameter; and terminal Windkessel boundary conditions modelling vascular beds. The model uses the nonlinear 1D equations of incompressible and axisymmetric flow in Voigt-type visco-elastic vessels (Alastruey *et al* 2011). It is based on the physical principles of conservation of mass, linear momentum and energy. The key assumptions used to model blood flow were laminar flow, incompressible and Newtonian blood (density, $\rho = 1060$ kg m^−3^, and viscosity, $\mu = 3.5$ mPa s), and no energy losses at bifurcations. We have previously shown that this approach is able to reproduce main features of arterial blood pressure and flow waveforms, by comparison against (i) *in vivo* data in rabbits and humans (Alastruey *et al* 2009, 2016), (ii) *in vitro* data in a 1:1 scale cardiovascular simulator rig of the aorta and its larger branches (Alastruey *et al* 2011), and (iii) numerical data obtained by solving the full 3D equations in compliant domains (Boileau *et al* 2015, Alastruey *et al* 2016). Further details of the model are provided in appendix A.

**Figure 1.**
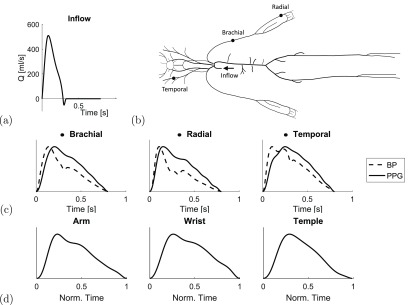
Simulating PPG pulse waves. (a) The baseline inflow waveform prescribed at the aortic root. (b) The arteries in the 1D numerical model, showing the sites of PPG pulse wave simulations: the radial, brachial and temporal arteries. (c) Estimating the PPG from blood pressure (BP). (d) *In vivo* PPG waves, for comparison.

The model configuration used in this study was as follows. The inflow waveform prescribed at the aortic root, shown in figure 1(a), was based on the waveforms reported in figure 3 of O’Rourke (2009). The waveform containing an inflection point on the systolic downslope was chosen, in keeping with the waveform used for pulse wave simulations in Mynard and Smolich (2015). The arterial tree contained 116 arterial segments, making up the larger arteries of the head, limbs and thoracic and abdominal organs as shown in figure 1(b). The geometry (lengths and radii) of the arterial segments were adapted from Mynard and Smolich (2015). The stiffness of each arterial segment was modelled following the approach in Mynard and Smolich (2015), using

1 \begin{align*} \newcommand{\e}{{\rm e}} \displaystyle \label{eqn:Eh} Eh = R_d \left[ k_1 \exp (k_2 R_d) + k_3 \right] \quad , \nonumber \end{align*}

where *E* is the Young’s modulus, *h* the wall thickness, *R*_*d*_ the diastolic radius, and $k_1 = 3.00 \times 10^6$ g s^−2^ cm^−1^, *k*_2_  =  −9 cm^−1^ and $k_3 = 3.37 \times 10^5$ g s^−2^ cm^−1^ are empirical constants. The resulting theoretical wave speeds, *c*_*d*_, can be obtained from *Eh* using

2 \begin{align*} \newcommand{\e}{{\rm e}} \displaystyle c_d = \sqrt{\frac{2Eh}{3\rho R_d}} \quad . \label{eqn:cd} \nonumber \end{align*}

**Figure 2.**
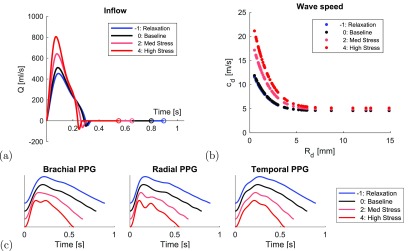
Simulating pulse waves during mental stress. (a) Prescribed aortic root inflow, *Q*, waveforms at baseline (black), relaxation (blue), and stress (red). (b) Prescribed wave speeds, *c*_*d*_, of each arterial segment of radius *R*_*d*_. (c) Simulated PPG waves at the brachial, radial and temporal arteries (offset for clarity).

The wave speeds of each of the arterial segments at baseline are shown in figure 2(b). Terminal branches of the arterial network were coupled to matched three-element Windkessel models to simulate the flow resistance and wall compliance of downstream vessels (Alastruey *et al* 2012). The total values of the resistance and compliance of each vascular bed were obtained from Mynard and Smolich (2015). The total values for each bed were split between each branch feeding into that bed by setting each branch’s Windkessel resistances and compliances respectively to be inversely proportional, and proportional, to the branch’s luminal area. The input parameters used in this study were selected to simulate a young, healthy adult (approximately 20–30 years of age, weight 75 kg, height 175 cm), as discussed in Mynard and Smolich (2015).

The model was extended in this work to simulate the PPG pulse wave. PPG waves were estimated from simulated blood pressure waves using the transfer function reported in Millasseau *et al* (2000) for estimating PPG waves from blood pressure waves at the finger. Further details of the transfer function are provided in appendix B. The blood pressure and PPG waves simulated at the three anatomical sites of interest (brachial, radial and right temporal arteries) are shown in figure 1(c). Corresponding *in vivo* PPG waves are shown for comparison in figure 1(d). These were acquired at the arm, wrist and temple from a healthy volunteer using a dedicated PPG sensor developed by Polar Electro (OH1 sensor, 135 Hz sampling frequency).

### 2.2. Simulating pulse waves during mental stress

Pulse waves were simulated at different levels of mental stress by adjusting the model input parameters to mimic haemodynamic changes which occur with stress. A literature review was performed to identify haemodynamic properties which change during mental stress. The changes described in each of the articles considered are detailed in table 1.

**Table 1.** Changes in haemodynamic properties induced by mental stress reported in the literature. Values are denoted as either mean (SD), or [lower, upper limit]. Definitions: HR: heart rate; SBP: systolic blood pressure; DBP: diastolic blood pressure; CO: cardiac output; SVR: systemic vascular resistance; PTT: pulse transit time; EF: ejection fraction; — not reported.

Study	No. Subjects	Age (yr)	HR (bpm)	SBP (mmHg)	DBP (mmHg)	CO (l min^−1^)	SVR (mmHg s ml^−1^)	PTT (ms)	EF (—)
Fauvel *et al* (1996)	20	42 (16)	15 (11)	15 (13)	10 (8)	—	—	—	—
Goldberg *et al* (1996)	196	62.5 (8.4)	10.1 (7.1)	30.5 (15.9)	11.7 (8.0)	1.0 (1.2)	0.02 (0.18)^e^	—	−4.5 (5.5)
Jain *et al* (1998)^a^	9	38 (11)	17 (11)	13 (8)	7 (8)	2.3 (1.7)	−0.09 (0.09)	—	4 (5)
Hey *et al* (2009)	12	[16, 19]	14.2 (19.2)	—	—	—	—	−22.9 (28.2)	—
Hjemdahl *et al* (1984)	12	—	28	29	14	4.6 (0.7)	−30%	—	—
Lindvall *et al* (1991)^a^	14	[21, 49]	16 (4)	20 (5)	14 (4)	1.5 (0.5)	−0.1 (0.1)	—	4 (3)
Lyu *et al* (2015)^b^	40	22 (1.7)	9.9 (18.3)	14.3 (16.2)	12.3 (10.6)	—	—	—	—
Sant’Anna *et al* (2003)^c^	18	28 (5)	5 (2)	7 (4)	13 (4)	—	—	—	4 (1)
Sawai *et al* (2007)	44	24.1 (4.6)	$[-7, 30]$	$[-10, 35]$	—	—	—	—	—
Ulrich *et al* (1991)	120	—	−2^d^	—	—	—	—	−4.8	—

Overall changes	na	na	[−8, 33]	[−10, 46]	[−1, 23]	[−0.2, 5.3]	[−0.2, 0.0]	[−51, 5]	[−1, 9]

a Data taken from their control groups.

b Data taken from their second level of task difficulty.

c Data taken from their placebo study.

d Assuming a baseline HR of 60 bpm.

e Data not used as obtained from patients with coronary artery disease, whose SVR tends to increase with stress, in contrast to the decrease observed in healthy subjects (Jain *et al* 1998).

All studies were carried out in healthy volunteers, except for the studies by Fauvel *et al* and Goldberg *et al* in which subjects with essential hypertension and stable coronary artery disease were used, respectively. The review indicated that the model input parameters affected by mental stress are: (i) the aortic root blood flow waveform (influenced by heart rate, HR, and cardiac output, CO), (ii) terminal resistances (determined by the systemic vascular resistance, SVR), and (iii) arterial stiffness (as indicated by changes in pulse transit time, PTT). In contrast, arterial geometry and blood properties were not found to be affected by mental stress. The overall changes of the haemodynamic properties affected by stress are provided in the final row of table 1. These overall changes were calculated from the lowest lower limits, and the highest upper limits, of the reported changes. Limits were derived from those values expressed as mean (SD) using: lower limit  =  mean—SD; upper limit  =  mean  +  SD.

The overall changes in model input parameters identified during the literature review were used to determine suitable model input parameters for simulations of PPG pulse waves during mental stress. Parameters were identified for six levels of mental stress (as detailed in table 2): baseline (i.e. no stress, level 0), increasing stress (levels 1 to 4), and decreasing stress (i.e. relaxation, level  −1). Suitable values of HR, CO and SVR were prescribed at each stress level to provide similar overall changes to those reported in the literature. Resultant SV values were derived using SV  =  CO/HR. Similarly, appropriate LVET (ms) values were derived from HRs (bpm) using an empirical relationship (Reant *et al* 2010):

3 \begin{align*} \newcommand{\e}{{\rm e}} \displaystyle \mathrm{LVET} = -1.5209 \, \mathrm{HR} + 375.96 \quad . \nonumber \end{align*}

**Table 2.** Haemodynamic properties of the model at different levels of mental stress. Definitions: AoE: aortic root Young’s modulus; AoPWV: aortic pulse wave velocity calculated using the foot-to-foot method from aortic root to aortic bifurcation. All other abbreviations are defined in table 1. SBP and DBP were calculated at the aortic root. PTT was calculated at the left digital artery.

Stress level	Prescribed properties			Resultant properties		Optimised properties			Measured properties	
	HR (bpm)	CO (l min^−1^)	SVR (mmHg s ml^−1^)	SV (ml)	LVET (ms)	SBP (mmHg)	DBP (mmHg)	PTT (ms)	AoE (kPa)	AoPWV (m s^−1^)
−1: Relaxation	66.9	5.2	1.03	78.2	307	105	70	133	335	4.7
0: Baseline	74.9	6.2	0.97	82.7	294	115	80	128	337	4.8
1: Stress	83.4	7.4	0.91	88.9	280	130	89	116	358	5.1
2: Stress	91.9	8.7	0.85	95.1	269	144	96	107	379	5.3
3: Stress	100.3	10.2	0.78	101.3	257	155	101	99	400	5.6
4: Stress	108.7	11.7	0.71	107.4	249	164	103	93	421	5.8
Overall changes	[−8, 34]	[−1.0, 5.5]	[−0.26, 0.06]	[−5, 25]	[−45, 13]	[−10, 49]	[−10, 23]	[−35, 4]	[−2, 84]	[−0.1, 0.9]

These cardiac properties were used to prescribe appropriate aortic inflow waveforms for the different stress levels, as shown in figure 2(a). The resulting inflow waveforms exhibited increased peak velocity and acceleration as expected due to the increase in ejection fraction during stress (Sabbah *et al* 1986). Arterial stiffness values were then optimised by modifying the values of *k*_1_ (controlling small artery stiffness) and *k*_3_ (controlling large artery stiffness) in equation (1) to produce similar ranges of the optimised properties (SBP, DBP and PTT, as specified in table 2) across the stress levels to those reported in the literature (specified in table 1). The resultant wave speeds, *c*_*d*_ (defined in equation (2)), of the arterial segments are shown in figure 2(b). The simulated PPG waves at different stress levels are shown in figure 2(c).

A further set of simulated pulse waves was created to analyse the physiological determinants of the PPG features. Each model input parameter (SV, HR, LVET, SVR, *k*_1_ and *k*_3_) was changed individually, taking the values listed in table 2, whilst all others were held at their baseline values. This resulted in a set of 31 simulations, consisting of the baseline case, and 30 additional simulations (one for each of the six model input parameters at each of the five stress levels).

### 2.3. Extracting PPG features

Feature measurements were extracted from the PPG pulse wave by identifying fiducial points on the pulse wave and its derivatives, and calculating a range of features from the fiducial points.

**Figure 3.**
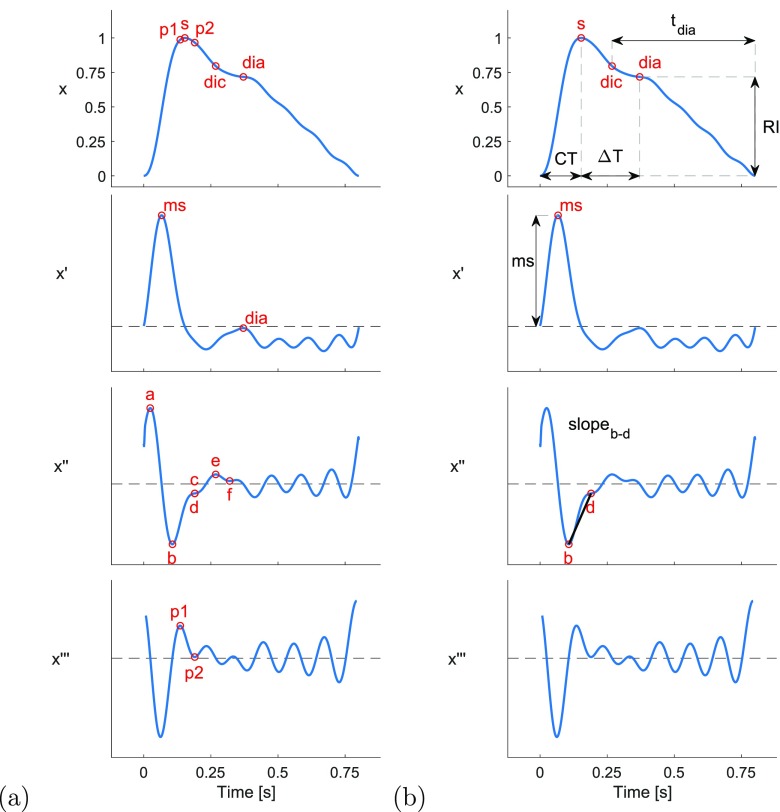
Extracting PPG features. (a) Detection of fiducial points on the PPG wave and its derivatives (defined in section 2.3). (b) Measurement of features from fiducial points (as defined in table 3).

The following fiducial points were detected: the systolic peak (s), dicrotic notch (dic) and diastolic peak (dia) in the pulse wave; the point of maximum upslope on the first derivative (ms); the a, b, c, d, and e waves in the second derivative (Elgendi 2012); and the early and late systolic components (p1 and p2) from the third derivative (Hayward and Kelly 1997). These points are illustrated for the baseline radial artery PPG pulse wave in figure 3. Details of the criteria used to detect these fiducial points are provided in appendix C.

**Table 3.** Features calculated from PPG pulse waves. Definitions: *t*—time since pulse onset (beginning of systolic upslope); *x*, $x'$, $x''$, $x'''$—PPG signal and derivatives; *T*—duration of cardiac cycle (s).

Signal	Approach	Feature	Formula	References
	Timings	$\Delta T$	$t(dia) - t(s)$	Chowienczyk *et al* (1999)
		*CT*	$t(s)$	Alty *et al* (2003)
		*prop*_*s*_	$t(s)/T$	Wu *et al* (2010)
		*t*_*sys*_	$t(dic)$	Ahn (2017)
		*t*_*dia*_	$T - t(dic)$	Ahn (2017)
		*t*_*ratio*_	$t(s) / t(dic)$	Ahn (2017)
		$prop_{\Delta T}$	$(t(dia) - t(s)) / T$	Ahn (2017)
		*t*_*p*1−*dia*_	$t(dia) - t(\,p1)$	Peltokangas *et al* (2017)
**PPG,** *x*		*t*_*p*2−*dia*_	$ t(dia) - t(\,p2)$	Peltokangas *et al* (2017)
		*IPR*	60/*T*	Lueken *et al* (2017)
	Amplitudes	*AI*	$(x(\,p2) - x(\,p1)) / x(s)$	Takazawa *et al* (1998)
		*RI*	$x(dia) / x(s)$	Chowienczyk *et al* (1999)
		*RI*_*p*1_	$x(dia) / x(\,p1)$	Peltokangas *et al* (2017)
		*RI*_*p*2_	$x(dia) / x(\,p2)$	Peltokangas *et al* (2017)
		*ratio*_*p*2−*p*1_	$x(\,p2) / x(\,p1)$	Peltokangas *et al* (2017)
	Areas	*A*1	area from pulse foot to dicrotic notch	Ahn (2017)
		*A*2	area from dicrotic notch to pulse end	Ahn (2017)
		*IPA*	*A*2/*A*1	Ahn (2017)

**PPG’**, $x'$	Amplitudes	*ms*	$x'(ms) / x(s) $	Alty *et al* (2003)

	Amplitudes	*b*/*a*	$x''(b)/x''(a)$	Takazawa *et al* (1998)
		*c*/*a*	$x''(c)/x''(a)$	Takazawa *et al* (1998)
		*d*/*a*	$x''(d)/x''(a)$	Takazawa *et al* (1998)
		*e*/*a*	$x''(e)/x''(a)$	Takazawa *et al* (1998)
		*AGI*	$(x''(b)-x''(c)-x''(d)-x''(e))/x''(a)$	Takazawa *et al* (1998)
**PPG”**, $x''$		*AGI*_*int*_	$(x''(b)-x''(e))/x''(a)$	Hyun *et al* (2008)
		*AGI*_*mod*_	$(x''(b)-x''(c)-x''(d))/x''(a)$	Ushiroyama *et al* (2005)
	Timings	*t*_*b*−*c*_	$t(c) - t(b)$	Ahn (2017)
		*t*_*b*−*d*_	$t(d) - t(b)$	Ahn (2017)
	Slopes	*slope*_*b*−*c*_	*d*/*dt* of straight line between *b* and *c*, normalised by *a*	Ahn (2017)
		*slope*_*b*−*d*_	*d*/*dt* of straight line between *b* and *d*, normalised by *a*	Ahn (2017)

Combined	multiple	*IPAD*	$(A2/ A1) + d/a$	Ahn (2017)
	Amplitudes	*k*	$x''(s) / ( (x(s)-x(ms)) / x(s)) $	Wei (2013)

A range of features were calculated from the fiducial points, as defined in table 3. These features were identified from publications describing techniques for assessing arterial stiffness from pulse waves. It seemed reasonable to expect these features to change during mental stress since arterial stiffness is greatly affected by mental stress.

### 2.4. Statistical analysis

PPG features which were indicative of mental stress were identified using the Mann–Kendall monotonic trend test, as described in Hamed (2008). This test was used (i) to assess whether each feature changed significantly at different levels of stress, and (ii) to quantify the strength of significant trends, as described in Kendall (1938). This analysis was performed using the set of six simulations representing the six stress levels. The strengths of trends were compared between measurement sites to determine whether any measurement site was more suitable for assessing stress.

The physiological determinants of PPG features were analysed using the set of 31 simulations in which model input parameters were changed individually whilst all others were held at their baseline values. The Mann–Kendall test was used to determine whether each PPG feature changed significantly when each input parameter was varied individually.

## 3. Results

### 3.1. Performance of PPG features for assessing stress

**Figure 4.**
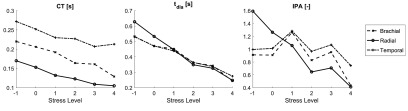
Changes in selected PPG features with stress.

The performances of the 32 PPG features were assessed at the three measurement sites. A total of 18 features exhibited significant trends with changes in stress at one or more of the measurement sites. The results relating to these features are provided in table 4. This table demonstrates that a wide range of features extracted from the PPG pulse wave and its first and second derivatives were indicative of mental stress. These were extracted from several different aspects of the signals: fiducial point timings and amplitudes, areas under the signals, and the slopes of lines joining fiducial points. The only aspect of the signals which did not result in any features indicative of stress was the analysis of PPG signal amplitudes. Three features were indicative of stress at all three measurement sites: *CT*, *t*_*dia*_ and *IPR*. These were extracted from timings of fiducial points on the PPG signal. The remaining 15 features listed in table 4 were only indicative of stress at one or two of the three measurement sites. Figure 4 shows the changes in three exemplary features with stress: *CT* and *t*_*dia*_, which significantly decreased with stress at all three sites; and *IPA*, which only decreased significantly at the radial artery.

**Table 4.** The PPG features which exhibited significant trends with changes in stress level (as defined in table 3). Definitions:  −trend: negative trend with increasing stress level;  +trend: positive trend;—: no significant trend.

Feature	Brachial	Radial	Temporal
*CT*	−trend	−trend	−trend
*prop*_*s*_	—	—	+trend
*t*_*dia*_	−trend	−trend	−trend
*t*_*ratio*_	—	−trend	—
$prop_{\Delta T}$	—	+trend	—
*IPR*	+trend	+trend	+trend
*A*2	—	−trend	−trend
*IPA*	—	−trend	—

*ms*	+trend	+trend	—

*b*/*a*	−trend	−trend	—
*c*/*a*	—	+trend	—
*AGI*	−trend	−trend	—
*AGI*_*inf*_	−trend	—	—
*AGI*_*mod*_	—	−trend	—
*t*_*b*−*c*_	—	—	−trend
*slope*_*b*−*c*_	+trend	+trend	—

*IPAD*	—	−trend	—
*k*	—	−trend	−trend

### 3.2. Comparison between PPG measurement sites

**Figure 5.**
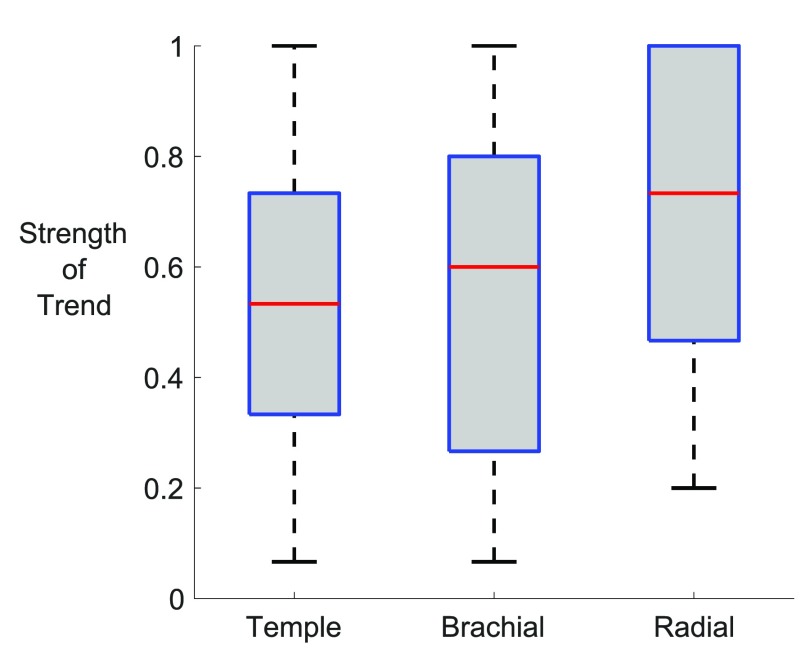
Box plots showing the distributions of the strengths of the trends of all 32 PPG features with stress at each of the three measurement sites. A strength of 0 indicates no trend, 1 indicates a strong trend, and a trend of  >0.86 was statistically significant (determined using the Mann–Kendall trend test). The strengths were significantly higher when measured at the radial artery than at the brachial or temporal arteries.

A greater number of PPG features were indicative of stress when measured at the radial artery (15 features), than when measured at the brachial (8) or temporal (7) arteries. Figure 5 shows the strengths of the trends with stress of all 32 PPG features at each site (including those which did not exhibit significant trends). The strengths were significantly higher when measured at the radial artery than at the brachial or temporal arteries. These observations indicate that the radial artery was the most suitable site for assessing the level of stress. There was no significant difference between the strengths of features measured at the brachial and temporal arteries.

### 3.3. Physiological determinants of PPG features

Table 5 shows the results of analyses of the physiological determinants of PPG features at each of the three measurement sites. The table indicates whether features increased (+) or decreased (−) significantly, or showed no significant trend (blank), when each model parameter was changed from its value at the minimum stress level (relaxation) to the maximum stress level whilst all others were held at baseline values. Most features were influenced by SVR, whereas relatively few were influenced by HR. *CT* and *t*_*dia*_ were influenced both by HR and by other parameters (including LVET at all sites, and SVR and SV at the radial and temporal sites). In contrast, features derived from the second derivative of the PPG were not influenced by HR, but by arterial stiffness.

**Table 5.** Statistically significant changes in PPG features when each model parameter was changed independently as occurs during increasing stress, whilst all others were held constant. Model parameters: *k*_3_ and *k*_1_ are constants determining large and small artery stiffness respectively (see equation (1)). Remaining model parameters and PPG features are defined in tables 2 and 3. Definitions: —: negative trend;  +: positive trend; all others non-significant.

Feature	Brachial						Radial						Temporal					
	*k*_3_	*k*_1_	SVR	LVET	HR	SV	k3	k1	SVR	LVET	HR	SV	k3	k1	SVR	LVET	HR	SV
*CT*				−	−				+	−	−	−		+	−	−	−	+
*prop*_*s*_				−	+				+	−	+	−		+	−	−	+	+
*t*_*dia*_	−	−	−	+	−		+		−		−	+				+	−	−
*t*_*ratio*_			−	+			+		+	−					−			
$prop_{\Delta T}$	−		+	−		−			+				+		+			
*IPR*					+						+						+	
*A*2			−			+	+		−	−	−	+	−		−	+		−
*IPA*	−		−	+		+	+		−		−	+	−		−	+		−
*ms*	+		−	+	+	+			−	+		+	−	−	+	+		−

*b*/*a*	−	−	+			−	−	−	+	−		−	+	+	−	−		+
*c*/*a*	+	+	+			−	+	+	−	+		+	−	−	−	+		+
*AGI*	−		+			+	−	−	+	−		−	+	+	−	−		
*AGI*_*inf*_	+		+	−		+	+	−		−		−	+	+	−	−		+
*AGI*_*mod*_	−	−	+			+	−	−	+			−	+	+	−	−		−
*t*_*b*−*c*_	+		−				+		−	−				−		−		
*slope*_*b*−*c*_	+	+					+	+		+		+		−		+		+

*IPAD*	+		−			+	+		−	−	−	+	−		−	+		−
*k*	+		−	−		+	+		+	−	−	−		+	−	−		+

The parameters which influenced particular PPG features varied between sites, providing insight into why certain features changed significantly with stress at some sites but not others. For instance, *slope*_*b*−*c*_ was positively influenced by arterial stiffness (*k*_1_ and *k*_3_) at the brachial site, and also by LVET and SV at the radial site. Indeed, it showed a positive trend with stress at these sites (see table 4). Conversely, *slope*_*b*−*c*_ was influenced negatively by *k*_1_ at the temporal site, which may explain why it did not show a significant trend with stress at this site.

The simulated PPG pulse waves used in these analyses are shown in the appendix D.

## 4. Discussion

In this study, we used a numerical model of pulse wave propagation to investigate the utility of a range of PPG features for assessing mental stress. PPG features were extracted from simulated PPG waves at three potential measurement sites. We identified three PPG features which were indicative of stress at all three measurement sites: the crest time (*CT*, time from pulse onset to peak), the duration of diastole (*t*_*dia*_, time from dicrotic notch to pulse end), and the instantaneous pulse rate (*IPR*). In addition, we found that a greater number of features were indicative of stress when measured at the radial artery than at the brachial or temporal arteries, indicating that PPG measurements at the radial artery may provide superior performance for assessing stress. Furthermore, we found that most individual features were affected by multiple cardiovascular properties, indicating that the changes in features with stress were the result of the collection of cardiovascular changes which occur during stress. These findings will be of use to device designers when considering how to process PPG signals, and where to measure PPG signals, to assess mental stress.

Several physiological mechanisms may have contributed to the changes in PPG features with stress. The decrease in *CT* was primarily due to the increase in HR and the decrease in LVET with stress. Both changes affected the shape of the blood flow waveform at the aortic root: they increased the slope in early systole and decreased the time of peak flow, thus decreasing *CT*. These aortic changes were transmitted to PPG measurement sites, where they were detected despite the disturbances introduced by wave reflections and viscous dissipation as the pulse wave propagates from the aortic root to measurement sites. The decrease in *t*_*dia*_ with stress was found to be related to the decrease in the time of the pulse end with increasing HR. Similarly, the decrease in *t*_*dia*_ also resulted in *A*2 (the area from dicrotic notch to pulse end) and consequently *IPA* (the ratio of diastolic to systolic areas) decreasing at the radial site. *CT*, *t*_*dia*_ and *IPA* may have particular utility as they do not depend on the absolute value of the PPG, so should be influenced less by the type of PPG sensor used.

Our approach for simulating PPG pulse waves using a numerical model of the circulation is novel. Previously PPG waves have been simulated by assuming that PPG morphology is linearly related to the luminal area of an artery (Epstein *et al* 2014). However, the PPG signal’s origins are complex, being related to not only blood volume, but also blood vessel wall movement, the orientation of red blood cells, and changes in capillary density (Allen 2007, Kamshilin *et al* 2015). Therefore, in this study we used an empirically-derived transfer function to estimate the PPG from arterial blood pressure. The transfer function was originally derived using data from normotensive, hypertensive, and vasodilated subjects, ensuring that it is suitable for use across a wide range of cardiovascular conditions (Millasseau *et al* 2000).

The use of a numerical model to simulate pulse waves at different levels of mental stress is also novel, to the best of our knowledge. The modelling of pulse waves during stress was performed by changing the model input parameters in accordance with the haemodynamic changes which occur with stress. Pulse waves were simulated at different levels of stress by incrementally changing the parameters from baseline values to the maximal changes reported in the literature. This ensured that the *in silico* simulations mirrored the changes which occur *in vivo* as closely as possible.

The methodology presented here for modelling the PPG pulse wave under different physiological conditions may be useful for identifying features of the PPG pulse wave which change during certain physiological changes. For instance, it has been widely suggested that a variety of features of the PPG pulse wave could be used to assess arterial stiffness (Millasseau *et al* 2006). Indeed, it has previously been proposed that the pulse wave can be used for cardiovascular disease classification (Elgendi 2012). In addition, it is reasonable to expect the pulse wave shape to change in many other pathophysiological conditions, since it is influenced by the cardiac, vascular, respiratory and autonomic nervous systems. The analysis of physiological determinants of PPG features presented in this study suggested that PPG features derived from the second derivative of the pulse wave are, in general, more strongly affected by arterial stiffness. This is in keeping with previous *in vivo* observations (Takazawa *et al* 1998), demonstrating how initial *in silico* studies could be used to inform *in vivo* studies.

There are several limitations to this study. Firstly, this was an *in silico* study. Consequently, further *in vivo* studies are required to determine how closely the simulated PPG waves compare with *in vivo* measurements, and to verify the observed changes in PPG features with stress. Secondly, only PPG features derived from a single pulse wave were assessed since the model did not account for lower frequency processes, such as inter-beat interval variability and respiration. Future studies should include metrics derived from multiple PPG pulse waves, such as inter-beat interval variability (Heilman *et al* 2008, Yoo and Lee 2011, Lee *et al* 2013, Zheng *et al* 2016), the surgical stress index (Huiku *et al* 2007, Abbod *et al* 2011, Lueken *et al* 2017), and the influences of respiration rate and depth (Sharma and Gedeon 2012). Thirdly, the transfer function used to estimate PPG signals from simulated blood pressure signals was designed using data acquired at the finger. Further work should investigate whether the use of individual transfer functions at different sites would improve PPG estimation. Fourthly, we have not assessed the potential effects of measurement noise on PPG features. Noise can be caused by movement artifact, optical interference, low perfusion levels and poor sensor contact (Karlen *et al* 2012). Measurement noise may impact features differently. Consequently, the relative utility of different features may not only depend on the strength of their changes with stress, but also on their robustness to noise. This is particularly important when using consumer devices to assess stress in daily life, when the influence of noise is likely to be greater than in the laboratory setting.

Future work should investigate improving techniques for assessing mental stress from the PPG. This study identified two pulse wave features which were particularly suitable for assessing stress. These were both derived from timings of fiducial points on the PPG signal. Several other features were also found to be indicative of stress, albeit not at all measurement sites. Many of these features were derived using other approaches, such as analysis of PPG pulse wave areas, and also from other signals including the first and second derivatives of the PPG. When assessing stress *in vivo* it may be beneficial to combine several features using a machine learning technique to provide a single, improved, indicator of stress. For instance, it may be beneficial to fuse the *CT* feature with *slope*_*b*−*c*_, since the former was found to be influenced by both LVET and HR (amongst others), whereas the latter was influenced by arterial stiffness, LVET and SV (at the radial site). This approach may improve performance in a heterogeneous population where some physiological changes are more pronounced than others in different subjects.

## 5. Conclusion

In this study we investigated the performance of several features of the PPG pulse wave for assessing mental stress. The main conclusion was that the crest time (time from pulse onset to peak) and the duration of diastole (time from dicrotic notch to pulse end) were identified as suitable candidates for assessing stress. These indices were influenced both by changes in heart rate and other cardiovascular properties. In addition, the analysis indicated that the radial artery was a more suitable measurement site for assessing stress than the brachial or temporal arteries. Further *in vivo* studies are required to verify these conclusions, and to develop an improved index of mental stress which combines several PPG features to provide improved performance. It may be beneficial to combine the crest time and duration of diastole with additional features derived from the second derivative of the PPG, since the latter were not influenced by HR.

## Figures and Tables

**Figure B1.**
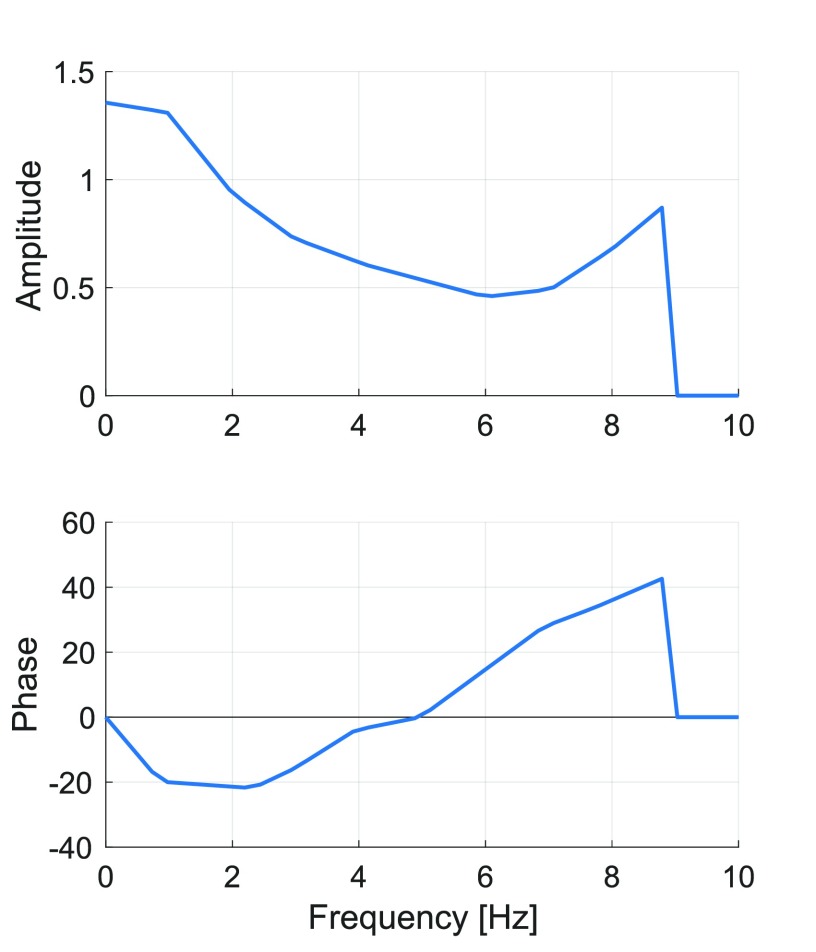
The transfer function used to estimate PPG pulse waves from blood pressure pulse waves.

**Figure D1.**
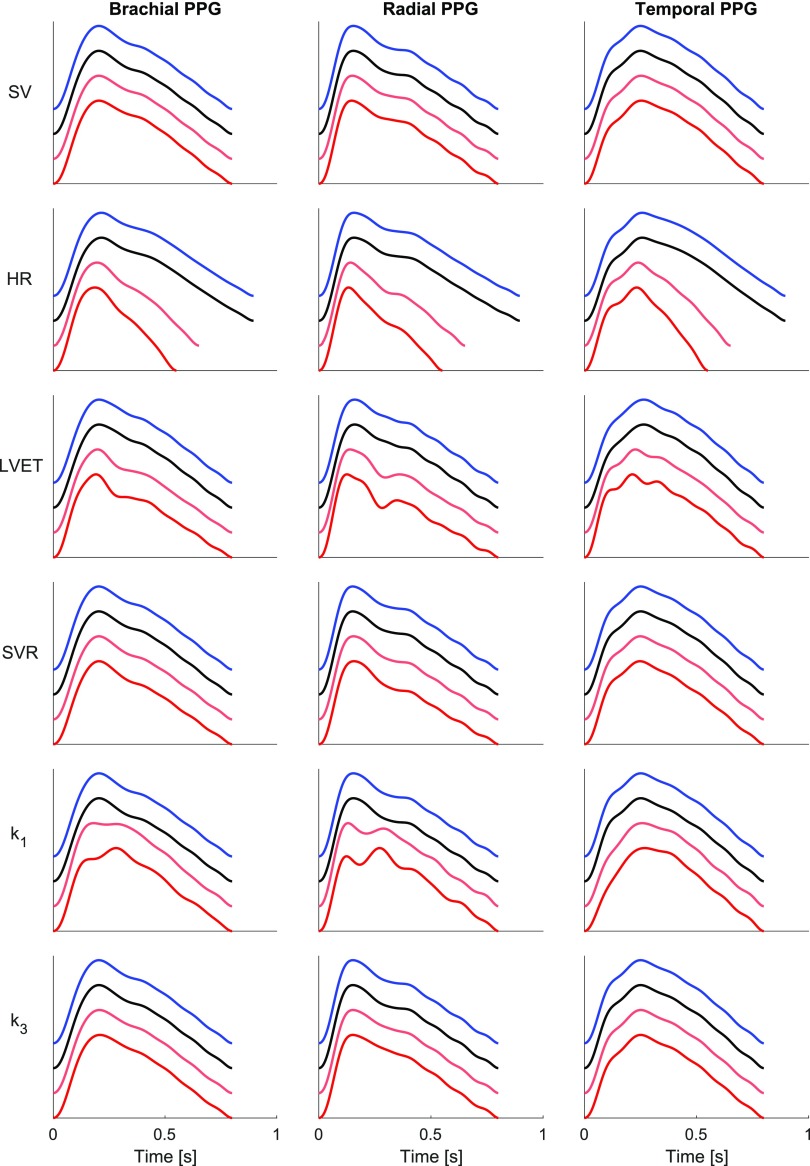
The simulated PPG pulse waves at baseline (black), and when each model input parameter was set to its relaxation value (blue), and increasing stress levels (light red, followed by dark red). All other input parameters were held at their baseline values. See table 5 for definitions.

## Data access statement

The data used in this research are provided as supplementary material (stacks.iop.org/PM/39/054001/mmedia) in Matlab ® format ($numerical\_ mental\_ stress\_ data.mat$). The simulated pulse waves generated by changing all model input parameters simultaneously are provided in the variable named $stress\_ levels\_ data$. The simulated waves generated by changing parameters individually are provided in the variable named $individual\_ params\_ data$. The data include (for each simulation, and each measurement site): simulated blood pressure (*P*) and PPG waves at the brachial, radial and temporal arteries (sampled at 1 kHz); pulse transit times (*PTT*) and pulse wave velocities ($PWV$) obtained using the foot-to-foot method from the aortic pressure wave; annotations of fiducial points on each pulse wave ($PPG.fid\_ pts$); and, features derived from each pulse wave ($PPG.cv\_ inds$). The script used to generate plots, and perform statistical analyses, is also provided ($mental\_stress\_numerical\_study\_analysis.m$). Further information about the data and conditions of access can be found by emailing research.data@kcl.ac.uk.

## Appendix A. Additional details of the numerical model of pulse wave propagation

Key details of the 1D model of pulse wave propagation were given in section 2.1. Additional details are provided in this section.

The governing equations of the model are described in Alastruey *et al* (2012): conservation of mass,

A.1 \begin{align*} \newcommand{\e}{{\rm e}} \displaystyle \frac{\partial A}{\partial t} + \frac{\partial (AU)}{\partial x} = 0 \quad , \nonumber \end{align*}

where *A* is the luminal area, *U* is the blood flow velocity, *t* is time, and *x* is the axial coordinate; and, conservation of momentum,

A.2 \begin{align*} \newcommand{\e}{{\rm e}} \displaystyle \frac{\partial U}{\partial t} + U \frac{\partial U}{\partial x} + \frac{1}{\rho} \frac{\partial P}{\partial x} = \frac{f}{\rho A} \quad , \nonumber \end{align*}

where $\alpha(x, t) = \frac{1}{AU} \int_{A} {\bf u}^2 d\sigma$ is the velocity profile shape factor, *P* is arterial pressure, $f(x, t)=2\mu \pi R \left[ \frac{\partial u}{\partial r}\right]_{r = R}$ is a frictional term, *ρ* is blood density, and *μ* is blood viscosity. The velocity profile was assumed to be close to plug flow in all arteries. The profile used was described in Alastruey *et al* (2012):

A.3 \begin{align*} \newcommand{\e}{{\rm e}} \displaystyle u(x,r,t) = U \frac{\zeta + 2}{\zeta} \left[1 - \left(\frac{r}{R}\right)^\zeta \right] \quad , \nonumber \end{align*}

where *r* is the radial coordinate, $R(x, t)$ is the radius of the lumen, and *ζ* is a constant. The profile satisfies the no-slip condition at the arterial wall (*u*|_*r*=*R*_  =  0). In this study, $\zeta = 9$.

In addition, a visco-elastic pressure–area relation (tube law) was used, given by

A.4 \begin{align*} \newcommand{\e}{{\rm e}} \displaystyle \label{eqn:tube_law} P = P_e(A, x) + \frac{\Gamma(x)}{A_d(x) \sqrt{A}} \frac{\partial A}{\partial t} \quad , \nonumber \end{align*}

where the wall viscosity coefficient, $\Gamma(x)$, is related to the wall viscosity, $\varphi(x)$, by $\Gamma(x) = \frac{2}{3} \sqrt{\pi} \varphi(x)h(x)$, and the elastic component of pressure, *P*_*e*_, is given by

A.5 \begin{align*} \newcommand{\e}{{\rm e}} \displaystyle \label{eqn:p_elastic} P_e(A,x) = P_d + \frac{\beta(x)}{A_d(x)} \left( \sqrt{A} - \sqrt{A_d(x)} \right) \quad , \nonumber \end{align*}

where subscript *d* denotes diastolic value, and

A.6 \begin{align*} \newcommand{\e}{{\rm e}} \displaystyle \beta(x) = \frac{4}{3} \sqrt{\pi} E(x)h(x) \quad , \nonumber \end{align*}

accounts for the stiffness of the arterial wall (Alastruey *et al* 2012), where $E(x)$ is the elastic modulus of the wall, and $h(x)$ is the wall thickness. In this study, *P*_*d*_  =  80 mmHg. $\Gamma (x)$ was modelled using the empirical relationship proposed in Mynard and Smolich (2015),

A.7 \begin{align*} \newcommand{\e}{{\rm e}} \displaystyle \Gamma(x) = \frac{b_1}{2R(x)} + b_0 \quad , \nonumber \end{align*}

where *b*_0_  =  400 g s^−1^, and *b*_1_  =  100 g cm s^−1^ as suggested in Mynard and Smolich (2015).

## Appendix B. Transfer function for estimating PPG from blood pressure

The transfer function used to estimated PPG pulse waves from arterial blood pressure pulse waves was reported in Millasseau *et al* (2000). It is an empirically determined transfer function, which has been shown to be able to reproduce digital blood pressure waves from digital PPG waves. Values for the amplitude and phase shift of the frequency response were obtained from figure 2(a) of Millasseau *et al* (2000). These values are given in table B1. The transfer function was implemented by calculating values for frequencies between 0 and 10 Hz using linear interpolation, as shown in the Bode plot in figure B1.

**Table B1.** The raw data which were interpolated to create the transfer function.

Amplitude		Phase (deg)	
Frequency (Hz)	Value	Frequency (Hz)	Value
0.00	1.36	0.00	0.00
0.97	1.31	0.87	−19.85
2.00	0.94	2.32	−21.84
2.97	0.73	2.97	−15.88
4.11	0.61	3.95	−3.97
6.00	0.46	4.97	0.00
7.03	0.49	6.92	27.79
8.00	0.68	7.73	33.75
8.97	0.91	8.92	43.68

## Appendix C. Identification of fiducial points

The criteria listed in table C1 were used to identify fiducial points on PPG pulse waves. The identified fiducial points were checked visually, and any inaccuracies were corrected manually.

**Table C1.** Criteria for identifying fiducial points on PPG pulse waves. Definitions: *x*, $x'$, $x''$, $x'''$—PPG signal and derivatives.

Signal	Fiducial point	Criterion
**PPG,** *x*	s	The maximum of *x*.
	*dic*	Coincident with *e*.
	*dia*	First local maximum of *x* after *dic* and before 0.8 T (or if no maxima then the first local maximum of $x''$ after *e* and before 0.8 T).

**PPG’**, $x'$	*ms*	The maximum of $x'$.

**PPG”**, $x''$	a	The maximum on $x''$ prior to *ms*.
	*b*	The first local minimum on $x''$ following *a*.
	*c*	The greatest maximum of $x''$ between *b* and *e* (or if no maxima then the first of (i) the first maximum on $x'$ after *e*, and (ii) the first minimum of $x'''$ after *e*).
	*d*	The lowest minimum on $x''$ after *c* and before *e* (or if no minima then coincident with *c*).
	*e*	The second maximum of $x''$ after *ms* and before 0.6 T (unless the *c* wave is an inflection point, in which case take the first maximum).
	*f*	The first local minimum of $x''$ after *e* and before 0.8 T.

**PPG”’**, $x'''$	*p*1	The first local maximum of $x'''$ after *b*.
	*p*2	Identify a candidate *p*2 at the last local minimum of $x'''$ before *d* (unless *c* = *d*, in which case take the first local minimum of $x'''$ after *d*). If there is a local maximum of *x* between this candidate and *dic* then use this instead.

## Appendix D. The effects of cardiovascular properties on simulated PPG pulse waves

The effects of changing individual cardiovascular properties on the simulated PPG pulse waves are shown in figure D1.
